# Using the knowledge-to-action framework with joint arthroplasty patients to improve the quality of care transition: a quasi-experimental study

**DOI:** 10.1186/s13018-020-1561-7

**Published:** 2020-01-29

**Authors:** Yaping Xu, Shuang Li, Peiyu Zhao, Jing Zhao

**Affiliations:** 1 Department of Orthopedics, China-Japan Friendship Hospital, Beijing, China; 2 Bone Necrosis and Joint Preservation Reconstruction Center, China-Japan Friendship Hospital, Beijing, China; 3 Department of Bone and Joint Surgery, China-Japan Friendship Hospital, 2 Yinghua Dongjie, Hepingli, Chaoyang District, Beijing, 100029 China; 4 Department of Nursing, China-Japan Friendship Hospital, Beijing, China

**Keywords:** Care transition, Quality, Quality improvement, Knowledge-to-action framework, Arthroplasty

## Abstract

**Background:**

Total joint arthroplasty is a mature and effective treatment for end-stage osteoarthritis. Assisting patients in completing the transition of the perioperative period and improving their satisfaction are important aspects of quality of care. This study aimed to investigate an intervention to improve the quality of care transition for joint arthroplasty patients informed by the knowledge-to-action (KTA) framework.

**Methods:**

In this quasi-experimental study, a total of 160 patients who underwent joint arthroplasty at a tertiary hospital from September to November 2018 and January to March 2019 were selected as participants using convenience sampling. The control group received routine medical care, while the observation group received medical care based on the KTA framework. Transitional care quality was assessed by the Care Transition Measure (CTM), with follow-up 1 week after discharge.

**Results:**

The observation group fared significantly better than the control group on general self-care preparation and written plan dimensions, as well as the quality of care transition. There was no significant difference in doctor–patient communication or health monitoring.

**Conclusions:**

The KTA framework provides a logical, valuable tool for clinical work. Using the KTA framework for joint arthroplasty patients helps to improve the quality of care transition, which is worth promoting.

## Background

Total joint arthroplasty is a common surgical procedure that reduces chronic joint pain and improves function as well as quality of life for older adults with osteoarthritis [1]. With the promotion of fast-track surgeries, the length of a hospital stay for joint arthroplasty patients is decreasing [2–4]. Assisting patients in completing the transition of the perioperative period, restoring function, and returning to normal life have become important directions for medical staff to explore.

The KTA framework was developed in Canada by Graham and colleagues in the 2000s, in response to the confusing multiplicity of terms used to describe the process of transforming knowledge to action [5]. This framework proposes a dynamic and iterative process consisting of two interacting phases: (1) the knowledge-creation cycle and (2) the action cycle. The knowledge-creation cycle consists of three phases and involves distilling knowledge from its most basic form to create a collection of synthesized, appraised, and user-friendly products catered to the needs of researchers and clinicians alike. The action cycle is the process of translating knowledge into practice comprising seven components: (1) identifying the problem, (2) adapting knowledge, (3) assessing barriers, (4) implementing, (5) monitoring, (6) evaluating, and (7) sustaining [6]. The application of this framework has significantly and positively influenced clinical practice and patient outcomes [6–10]. To our knowledge [11], there are few reports on the application of the KTA framework in China; use of the framework for joint arthroplasty patients has not been explored.

The aim of this study was to investigate the effect of the KTA framework to improve the quality of care transition for joint arthroplasty patients. It is worth mentioning that we used a questionnaire with good reliability and validity for evaluation, which ensured the authenticity of the implementation effect of our medical care. Furthermore, our work serves as a reference point for the consistent improvement of care transition and contributes to the evidence base guiding the formulation of health strategies by medical institutions.

## Methods

### Design and setting

**Fig. 1:**
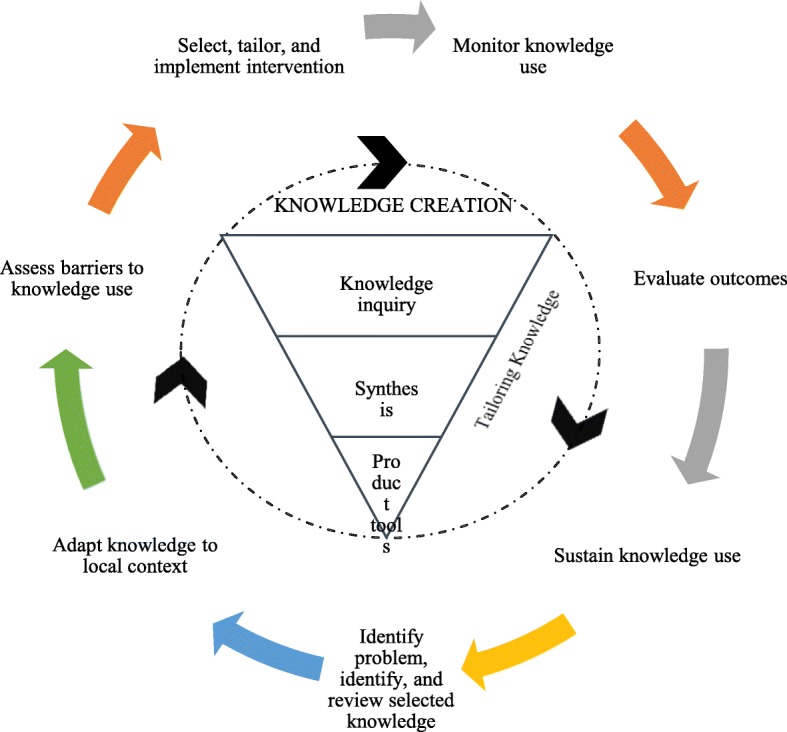
The knowledge-to-action cycle, adapted from the model by Graham and colleagues [5], with specific measures shown below

The study followed a quasi-experimental design. A total of 160 patients who underwent joint replacement at a tertiary hospital from September to November 2018 and January to March 2019 were selected as the study participants using convenience sampling. A total of 141 patients responded to the survey (response rate, 88.1%). The demographic characteristics of the participants are shown in Table 1. Demographic data of both groups included gender, age, highest education level, marital status, and direct family members; the differences were not statistically significant (*P* > 0.05). Patients from the first cohort were included in the control group, and patients from the second cohort were included in the observation group. The control group received routine medical care, while the observation group received medical care based on the KTA framework (Fig. 1). Transitional care quality was assessed by the Care Transition Measure (CTM) and followed up on 1 week after discharge.

**Table 1:** Characteristics of the respondents (*N* = 141)

Characteristic	Category	CG (*N* = 90)	OG (*N* = 51)	*χ*^2^	*P*
Gender				0.086^a^	0.769*
	Male	34	18		
	Female	56	33		
Age (years)				0.488^b^	0.936*
	< 50	11	6		
	50–59	22	15		
	60–69	33	17		
	70–79	24	13		
Highest education attained				6.124^b^	0.050*
	Junior high school or below	46	37		
	Technical secondary school/high school	32	11		
	College or above	12	3		
Marital status					
	Married	86	46	1.965^b^	0.417*
	Unmarried	1	1		
	Widowed	3	4		
Direct family members					
	Spouse	63	29	3.467^b^	0.505*
	Living alone	1	1		
	Nanny	2	2		
	Children	20	17		
	Others	4	2		

*P* values calculated using the *χ*^2^ method

*CG* control group, *OG* observation group, *TKA* total knee arthroplasty, *UKA* unicompartmental knee arthroplasty, *THA* total hip arthroplasty

^a^Pearson’s chi-square

^b^Fisher’s exact test

*No significant difference among groups (*P* > 0.05)

The CTM was designed to assess the effectiveness of interventions aimed at improving care transitions [12], and it is a valid and reliable tool used for measuring the quality of care transition [13, 14]. A Chinese version (CTM-C), with reliability and validity established, is used to assess the quality of transitional care in mainland China [15]. The content validity index for the CTM-C’s total scale is 0.99. It is composed of 17 items, each using a 4-point Likert scale ranging from “strongly disagree” to “strongly agree.” Factor analysis revealed four dimensions—general self-care preparation, written plan, doctor–patient communication, and health monitoring—with a cumulative variance of 58.96%. The Cronbach’s α for the total scale was 0.85, while that for each factor varied from 0.61 to 0.89 [15]. Finally, the average score of the items in the percentage system was used as the scale score, and the following conversion formula was used: scale score = (average score of items − 1) 100/3. The score ranged from 0 to 100, with a higher score indicating an improvement in the quality of care transition in the hospital [16].

Two trained volunteers conducted telephone follow-up to evaluate the quality of care transition provided by the medical staff during hospitalization, and it was conducted in two parts. The first part involved the collection of demographic data, while the second involved the administration of the CTM-C to assess the quality of care transition.

The study was approved by the institutional review board of the China-Japan Friendship Hospital and adhered to the Helsinki Declaration. Participants’ written informed consent was not required because the questionnaire was anonymized and posed less than minimal risk.

### Data analysis

Descriptive statistical analysis was conducted to summarize the participants’ demographic characteristics. Categorical data were reported as frequencies. Comparisons were conducted using the Pearson chi-squared test. Fisher’s exact test was used for 2 × 2 tables where more than 25% of the expected counts were less than five for the chi-squared test. The Mann–Whitney *U* test was implemented as the nonparametric test of the two independent samples. The differences in the four dimensions and overall quality of care transition were also analyzed using the Mann–Whitney *U* test. A two-tailed *P* value of < 0.05 was considered statistically significant. All data analyses were performed using IBM SPSS Statistics for Windows version 19.0 (IBM Corp., Armonk, NY, USA).

## Results

### Comparison of the quality of care transition between the two groups

As shown in Table 2, the observation group receiving medical care based on the KTA framework fared significantly better than the control group in the dimensions of general self-care preparation and written plan; the difference was statistically significant (*P* < 0.05). The quality of care transition in the observation group was better than that of the control group, and this difference was statistically significant (*P* < 0.05). There was no significant difference in the dimensions of doctor–patient communication and health monitoring between the two groups (*P* > 0.05).

**Table 2:** Comparison of the quality of care transition between the two groups with joint arthroplasty via the CTM-C

Groups	Number	Dimension 1 (mean rank)	Dimension 2 (mean rank)	Dimension 3 (mean rank)	Dimension 4 (mean rank)	Total factor (mean rank)
CG	90	64.55	58.81	66.58	67.45	63.56
OG	51	82.38	92.51	78.80	77.26	84.14
*Z*		− 2.537	− 4.872	− 1.839	− 1.394	− 2.882
*P*		0.011*	0.000*	0.066	0.163	0.004*

Dimension 1, general self-care preparation; dimension 2, written plan; dimension 3, doctor–patient communication; dimension 4, health monitoring

*P* values calculated using the Mann–Whitney *U* test

*CTM-C* The Chinese version of Care Transition Measure, *CG* control group, *OG* observation group

*Significant difference among groups (*P* < 0.05)

## Discussion

### Effect of using the KTA framework to improve the quality of care transition of joint arthroplasty patients

Our findings indicate that using the KTA framework with joint arthroplasty patients improved the quality of their care transition. Patients gave a good evaluation for the overall quality of care transition (84.14). Although there was no significant influence on the doctor–patient communication and health monitoring dimensions, our findings suggest that the medical care based on the KTA framework could help to improve the quality of care transition due to the contribution of the general self-care preparation and written planning dimensions. In terms of scores alone, each dimension showed an improvement in the observation group compared to the control group. The application of the KTA framework has significantly and positively influenced clinical practice. There is still room for the improvement in quality of care transition, which needs further exploration in research and by the medical staff.

### Focus on the quality of care transition of patients with joint arthroplasty

Our findings further suggest medical staff need to focus on the quality of care transition of patients with joint arthroplasty. As shown in Table 2, the participants in the control group gave a poor evaluation to general self-care preparation, written plan, doctor–patient communication, and health supervision dimensions, with each dimension scoring below 70. The overall evaluation of the quality of care transition was also low (63.56). Compared to developed countries, research into care transition started later and developed slowly in China. Most studies pay attention to patients’ needs for care transition, as well as exploring modes of care transition [17], while paying less attention to the evaluation of the quality of care transition. Although the current study was conducted in a tertiary hospital, our findings may also hold value for other types of medical institutions.

### Clinical applications of the KTA framework

In this study, we first identified the problem based on the results of the control group, after which we carried out knowledge retrieval, integration, and output and localized the knowledge. As shown in Fig. 1, the steps included assessing the barriers to knowledge use, developing practical interventions for the control group, and monitoring knowledge use. The outcomes were assessed using CTM-C. In addition, knowledge was maintained and utilized. The KTA framework provides a logical, valuable tool for clinical practice. Through this, we were able to make meaningful conclusions that could be used as a basis for future studies. This is, to our knowledge, the first study using the KTA framework, along with a questionnaire with good reliability and validity, to improve the quality of care transition for joint arthroplasty patients; however, our study does have some limitations. First, participants were purposively selected from patients with joint replacement in the orthopedic department of a level-three, class-A hospital. Non-random grouping affects the reliability of research results to some extent. Considering this, we analyzed the demographic data of the two groups of participants; the differences were not statistically significant (*P* > 0.05). Second, the sample size was not large enough, which may limit the generalizability of our results.

## Conclusion

With the advent of the era of fast-track surgery, the quality of care transition for patients undergoing joint replacement is worthy of attention. The KTA framework provides a logical, valuable tool for clinical practice. Using the KTA framework with joint arthroplasty patients helps to improve the quality of care transition, which is worth promoting.

## Data Availability

Data collected from the survey were anonymized. The raw data from which the paper’s results were derived can be made available on request.

## Abbreviations

CTM: Care Transition Measure
CTM-C: Care Transition Measure – Chinese Version
KTA: Knowledge-to-action
